# Enhanced early skin cancer detection through fusion of vision transformer and CNN features using hybrid attention of EViT-Dens169

**DOI:** 10.1038/s41598-025-18570-1

**Published:** 2025-10-06

**Authors:** Hanan T. Halawani, Ebrahim Mohammed Senan, Yousef Asiri, Ibrahim Abunadi, Aisha M. Mashraqi, Eman A. Alshari

**Affiliations:** 1 Department of Computer Science, College of Computer Science and Information Systems, Najran University, 61441 Najran, Saudi Arabia; 2 Department of Artificial Intelligence, Faculty of Computer Science and Information Technology, Al-Razi University, Sana’a, Yemen; 3 Department of Computer Science, College of Applied Sciences, Hajjah University, Hajjah, Yemen; 4 Department of Software Engineering, College of Computer and Information Sciences, King Saud University, P.O. Box 103786, 11543 Riyadh, Saudi Arabia; 5 Computer Science and Information Technology Department, Thamar University, Dhamar, Yemen

**Keywords:** Deep learning, Enhanced ViT-Encoder, Enhanced DenseNet169, Skin cancer, Hybrid model, Cancer, Computational biology and bioinformatics, Engineering, Mathematics and computing

## Abstract

Early diagnosis of skin cancer remains a pressing challenge in dermatological and oncological practice. AI-driven learning models have emerged as powerful tools for automating the classification of skin lesions by using dermoscopic images. This study introduces a novel hybrid deep learning model, Enhanced Vision Transformer (EViT) with Dens169, for the accurate classification of dermoscopic skin lesion images. The proposed architecture integrates EViT with DenseNet169 to leverage both global context and fine-grained local features. The EViT Encoder component includes six attention-based encoder blocks empowered by a multihead self-attention (MHSA) mechanism and Layer Normalization, enabling efficient global spatial understanding. To preserve the local spatial continuity lost during patch segmentation, we introduced a Spatial Detail Enhancement Block (SDEB) comprising three parallel convolutional layers, followed by a fusion layer. These layers reconstruct the edge, boundary, and texture details, which are critical for lesion detection. The DenseNet169 backbone, modified to suit dermoscopic data, extracts local features that complement global attention features. The outputs from EViT and DenseNet169 were flattened and fused via element-wise addition, followed by a Multilayer Perceptron (MLP) and a softmax layer for final classification across seven skin lesion categories. The results on the ISIC 2018 dataset demonstrate that the proposed hybrid model achieves superior performance, with an accuracy of 97.1%, a sensitivity of 90.8%, a specificity of 99.29%, and an AUC of 95.17%, outperforming existing state-of-the-art models. The hybrid EViT-Dens169 model provides a robust solution for early skin cancer detection by efficiently fusing the global and local features.

## Introduction

One of the most common cancer types across the world, skin cancer is caused in the greater part by ultraviolet (UV) radiation from sunlight^1^. Common forms of skin cancer include basal cell carcinoma (BCC), squamous cell carcinoma (SCC), and malignant melanoma, which are aggressive because of their ability to rapidly metastasize^2^. Although various treatments, from surgical excision, radiation therapy, and immunotherapy, are available, detection remains an important factor in decreasing early death rates^3^. Traditionally, the diagnosis of skin cancer was based on visual examination by a dermatologist, with dermatoscopic imaging sometimes used as support to magnify the features and structures of the skin that might be invisible to the naked eye. Dermatoscopy enables dermatologists to observe patterns, colors, and textures characteristic of a group of skin lesions that are distinct from others, thereby improving diagnostic accuracy^4^. However, several constraints exist in manual diagnosis with dermatoscopes. First, many early stage malignant lesions are often misdiagnosed as benign because of their acute visual similarity^5^. There is a significant degree of variability in doctors’ diagnoses, particularly among general practitioners with limited dermatological training. The shortage of experienced dermatologists is a considerable impediment to providing proper and timely evaluations, especially in rural or resource-constrained settings^6^. In recent years, artificial intelligence systems, particularly deep-learning models, have emerged as tools for medical image analysis^7^. Deep learning models have superior capabilities for analyzing massive datasets of skin images, independently learning patterns that indicate different skin conditions^8^. These systems assign consistent evaluations, reduce subjective interpretations, and expand the diagnostic range. CNNs are highly effective for feature extraction owing to their ability to extract hierarchical features. These multiple convolutional and pooling layers enable convolutional neural networks to capture low- or high-frequency features^9^. The initial layers extract basic shapes and textures such as color and texture^10^. In contrast, deeper layers extract complex features, such as irregular lesion borders, asymmetry, and pigmentation abnormalities, which are necessary for dermatological diagnosis. However, CNNs are limited to extracting local features^11^. To address this issue, ViT models have been introduced^12^. ViTs divide an image into a series of patches without overlaps, attaching to their line-like embeddings fed into self-attention mechanisms, unlike CNNs^13^. This ability enables ViT models to capture global spatial associations, thereby accounting for long-distance dependencies and subtle structural variations throughout the image under scrutiny. The complementary nature of CNN and ViT techniques has led to the development of hybrid models^14^. These structures combine the ability of CNN techniques to capture fine-grained local features with the strength of ViT techniques in modeling the global context. By combining both methods, hybrid models offer a more comprehensive view of lesions, thereby improving classification performance in similar diagnostic cases^15^.

Main contributions of this study.

- This work presents several key advancements in automated skin lesion analysis for the early detection of cancer. First, we developed an encoder image enhancement pipeline for the ISIC 2018 dataset, combining an Encoder Adaptive Average Filter Encoder for noise reduction with Encoder Laplacian sharpening to enhance edge details, followed by a dedicated encoder hair removal algorithm to improve the feature visibility in dermoscopic images.
- Computational efficiency is significantly improved by optimizing the DenseNet169 architecture through a strategic reduction of convolutional layers while maintaining feature extraction capability, thereby lowering computational costs without compromising diagnostic accuracy.
- To address the inherent limitations of Vision Transformers in medical imaging, an Encoder SDEB block within the ViT framework is introduced. The encoder recovers fine-grained spatial information lost during patch segmentation, preserving the critical lesion boundaries and texture patterns.
- The core innovation lies in the encoder hybrid EViT-Dens169 architecture, which synergistically combines the enhanced encoder (featuring six attention blocks with expanded Multi-Head Self-Attention) with the optimized Encoder DenseNet169 backbone. This integration enables simultaneous modeling of the encoder global contextual relationship encoder through transformer attention mechanisms and the encoder local feature extraction encoder via convolutional operations, providing comprehensive lesion analysis.

The remainder of this paper is organized as follows. Section “Related work” presents a review of the methodologies and results of related studies in the field of dermoscopic image analysis and skin lesion classification. Section “Materials and tools” describes the materials and tools used in the study. Section “Results” provides an in-depth analysis of the results obtained using the proposed hybrid model. Section “Comparison and discussion of systems analysis” presents a comparative evaluation of the proposed system with those of previous studies and discusses its significance. Finally, section “Conclusions” summarizes the conclusions of this study.

## Related work

In this section, analyze the methodologies, architectures, and results of previous studies focused on the diagnosis of skin cancer using deep learning approaches.

Houssein et al.^16^ presented a custom-designed DCNN for classifying skin cancer lesions using the ISIC 2018 dataset, which is notably imbalanced. The reported gains are primarily in accuracy, with limited insight into how the model mitigates class imbalance beyond raw metric improvement. Additionally, comparisons often rely on baseline models without fine-tuning the details, which can potentially underrepresent their actual capabilities. Sivakumar et al.^17^ presented an automated diagnostic framework for the detection of malignant melanoma by integrating a CNN-based model with ResNet-50 and a supporting web application. The methodology involves a sequential pipeline of data preprocessing, noise reduction, image enhancement, segmentation, feature extraction, and hybrid pooling strategies to refine classification. Critically, while the model reports an accuracy of 94% and an F1-score of 93.9%, the framework lacks a detailed comparison with fine-tuned state-of-the-art models and does not clarify the robustness of the system across diverse image conditions or class imbalance. Sabir et al.^18^ explored melanoma classification using three individual CNN architectures. The methodological pipeline includes image normalization, augmentation, segmentation, and classification. However, this approach is limited by its reliance on isolated architectures. The system does not explore feature integration across multiple CNNs or between CNNs and transformer-based models such as ViT, which could potentially enhance robustness. Although EfficientNet-B0 performed well, its standalone use may restrict the model’s generalizability across diverse image types and complex lesion morphologies. Musthafa et al.^19^ presented a custom CNN model for classifying skin lesions using the ISIC 2018 dermoscopic image dataset. The architecture comprises multiple convolutional, pooling, and dense layers, with data augmentation techniques applied to address the class imbalance. This methodology is constrained by its reliance on a single-CNN framework. It does not explore hybrid architectures such as CNN-ViT integration, nor does it fuse features from multiple CNNs, which can enhance feature diversity. These limitations suggest missed opportunities to further improve the generalizability and robustness of the diagnostics. Flosdorf et al.^20^ investigated skin cancer detection using two ViT models and averaged self-attention mechanisms to enhance the lesion classification. Both pre-trained ViT variants were evaluated against classical classifiers (KNN, Decision Tree) and CNN baselines. Despite the use of the ViT models, the methodology remains limited. There is no integration of CNN and ViT features to exploit both local and global patterns, nor any fusion of multiple CNNs to enhance the discriminative power. Abdullah et al.^21^ introduced a two-stage methodology for melanoma classification using a custom deep sequential CNN. In stage one, the model performs preprocessing to isolate the lesion regions and extract key visual features. The model was trained, validated, and tested on the ISIC 2018 dataset, achieving classification accuracies of 86% (InceptionV3), 90% (ResNet50 + VGG16), and 92.14% (ViT). This study has several methodological limitations. The model does not integrate features from multiple CNN architectures, which can enhance robustness. It also lacks hybridization with ViTs and lacks the opportunity to combine spatial (CNN) and global (ViT) representations. Additionally, no feature-level fusion or optimization of deeper CNNs was conducted. Gamage et al.^22^ presented a melanoma classification framework combining CNN and ViT architectures utilizing the ISIC 2018 dataset. A U^2^-Net segmentation model generates lesion masks, guiding both CNN- and ViT-based pipelines. Grad-CAM and Grad-CAM +  + were employed to interpret the model decisions using heatmaps. The ViT-based approach achieved an accuracy of 92.79%, a sensitivity of 91.09%, and a specificity of 93.54%. The system addresses CNNs and ViTs separately without exploring hybrid architectures that fuse the CNN and transformer features, which is an increasingly effective direction. These gaps highlight opportunities for methodological enhancement and generalization. Swetha et al.^23^ investigated multiclass skin lesion classification using transfer learning with eight pre-trained CNN architectures. The method reported a categorical accuracy of 83.69% and Top-2 accuracy of 91.48%. First, it does not explore feature-level integration across multiple CNNs, which can enhance robustness. Additionally, there has been no effort to optimize or adapt deeper CNN layers or architectures to the domain-specific characteristics of dermoscopic images. Kandhro et al.^24^ presented binary skin lesion classification (malignant vs. benign) using multiple pre-trained CNN architectures. An enhanced VGG19 (E-VGG19) was proposed by appending additional max-pooling and dense layers. The extracted features from these deep models were then classified using traditional machine learning algorithms. The architecture lacks integration with modern attention-based models, such as ViT, which have demonstrated superior performance in recent literature. In addition, no optimization or pruning strategies were applied to the deeper CNNs, potentially leading to inefficiencies. Balaha et al.^25^ presented a hybrid framework for skin lesion segmentation and classification, combining U-Net-based automatic segmentation, manual annotation, and hyperparameter optimization using the Harris Hawks Optimization (HHO) algorithm. Five pre-trained CNNs were optimized via HHO and applied to the two datasets. The segmentation phase leverages U-Net, where masks are unavailable, reporting a solid performance (e.g., 91.95% accuracy, 0.159 loss). The method relies on single-model pipelines rather than feature fusion across multiple CNNs, which can improve generalization. Havirbhavi et al.^26^ explored melanoma detection using two CNN models: a standard ResNet50 for feature extraction and a custom CNN for classification. The models were optimized using the Adam optimizer and categorical and cross-entropy losses. A TQDM-based progress bar was used to monitor training convergence. Although this approach effectively applies standard techniques, several methodological limitations are evident. The method does not explore multi-CNN feature fusion, nor does it integrate CNN features with ViT representations, which can provide both local and global contextual understanding. Adebiyi et al.^27^ utilized the ISIC 2018dataset to train a multimodal deep learning model that integrates both dermoscopic images and patient metadata (age, sex, lesion site). The data were split into 70%, 20%, and 10% for training, validation, and testing, respectively. The multimodal model was benchmarked against image-only CNN-based models using accuracy and AUC-ROC as the evaluation metrics. The multimodal model achieved promising results with 94.11% accuracy and 0.9426 AUC. The model lacks optimization of deep features via dimensionality reduction or metaheuristic methods. Toprak et al.^28^ presented a hybrid model utilizing DeepLabV3 + for lesion segmentation and features extracted from three CNNs, followed by Relief-based feature selection and KNN classification. Although the model achieved 94.42% accuracy on ISIC-2019, it failed to optimize or deeply fuse CNN outputs for enhanced performance. Courtenay et al.^29^ employed near-infrared hyperspectral imaging (900.6–1454.8 nm) to classify lesions in 125 patients across three NMSC types, using a hybrid CNN for feature extraction and an SVM for classification. The model struggled to distinguish between malignant classes while achieving an accuracy of over 80%. The methodology failed to transfer learning from shorter wavelengths to enhance the performance, indicating limited cross-spectral generalizability. Thapar et al.^30^ presented a skin cancer detection framework using dermoscopy images, where lesion segmentation is performed via Grasshopper Optimization and ROI localization is guided by Swarm Intelligence. SURF was employed for handcrafted feature extraction, and its classification was evaluated. The absence of deep-learning-based feature optimization or hybridization with radial or spectral data limits its scalability and modern relevance. Faghihi et al.^31^ presented a CNN-based approach to melanoma classification by integrating VGG16 and VGG19 into a modified AlexNet architecture. This approach lacks fusion with the ViT optimization of deeper CNNs, limiting potential improvements in generalizability and robustness. Tai et al.^32^ introduced a lightweight deep neural network using Double-Condensing Attention Condensers (DC-AC) within a self-attention backbone to detect skin cancer from lesion images. The model achieved AUCs of 0.90. However, it does not explore the optimization of larger models for comparative scalability or interpretability.

It should be noted that previous studies presented notable limitations, including reliance on isolated CNN or ViT models without integrating their complementary strengths. Many omit feature fusion across multiple CNNs, ignore hybrid CNN-ViT architectures, or fail to optimize deep CNN features for lesion diversity and class imbalance. This study addresses these gaps by proposing a hybrid framework that integrates the CNN and ViT models, enabling both local and global feature learning. Moreover, feature-level fusion and model optimization strategies were incorporated to enhance the classification robustness, generalizability, and clinical relevance.

## Materials and tools

**Fig. 1:**
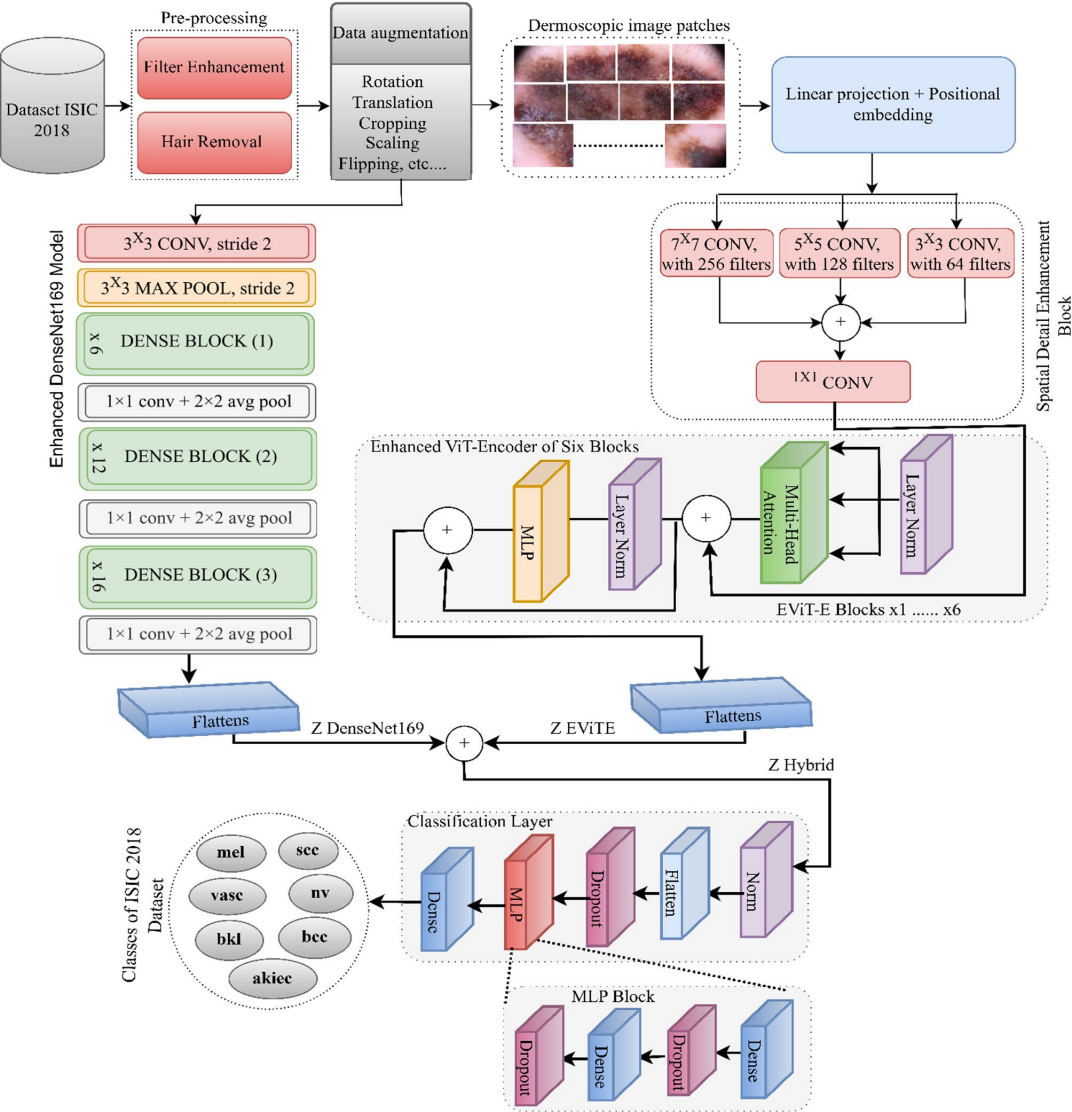
Framework for the hybrid EViT-DenseNet169 methodology for analyzing dermoscopy images for early skin cancer classification.

The workflow in Fig. 1 begins with the ISIC 2018 skin lesion dataset, and input images undergo preprocessing steps, such as filter enhancement and hair removal, to make the lesion more visible. These are followed by data augmentation techniques, such as rotation, scaling, and flipping, to make the model more robust and generalizable. The preprocessed and augmented images are passed through a series of convolutional layers, which preserves the spatial dimensions and extracts the initial features. This was followed by three DenseNet blocks. Each dense block was followed by a 1 × 1 convolution and a 2 × 2 average pooling operation to progressively downsample the features while retaining critical information. In parallel, the images were also processed through the enhanced DenseNet169 model, where the layers and filters were reduced to reduce the computational process. The outputs were summed and passed through a 1 × 1 convolution to unify the channel dimensions. This branch is followed by linear projection and positional embedding to prepare inputs for the transformer blocks. Both feature streams (from DenseNet and the transformer preparation path) are flattened and fed into the Enhanced EViT Encoder module, which contains six blocks, each consisting of an MLP, Layer Normalization, and Multi-Head Self-Attention. These EViT Encoder blocks capture the global and contextual relationships among the features. The outputs from both branches are concatenated and passed to a classification head composed of MLP blocks, dropout layers, and flattening and normalization layers. The final classification layer outputs predictions for the seven classes of skin lesions. This dual-stream fusion of CNN and ViT provides both local texture-level and global semantic-level understanding, thereby enhancing the classification accuracy for complex skin lesion images.

### Description of dermoscopy ISIC-2018 datasets

The ISIC 2018 dataset is an epoch-making collection of dermatological imagery and computer-aided diagnoses of skin lesions. It is among the largest publicly available databases of dermoscopic images and was comprehensively developed through the collaborative efforts of international medical institutions, including the Medical University of Vienna’s Department of Dermatology and Queensland’s Australia Skin Cancer Clinic. Comprising 10,015 high-quality dermoscopic images, the ISIC 2018 dataset presents researchers with a difficult benchmark for developing and evaluating diagnostic algorithms. The images were categorized into seven classes that encompassed both melanocytic and non-melanocytic lesions. The database contains 6,705 images of Melanocytic Nevi (NV), 1,113 images of melanoma (Mel), 1,099 images of Benign Keratosis Lesions (BKL), 514 images of Basal Cell Carcinoma (BCC), 327 images of Actinic Keratoses (AKIEC), 142 images of Vascular Lesions (VASC), and 115 images of dermatofibroma (DF)^33^. The distribution also reflects the natural prevalence of these conditions in clinical practice; however, it introduces a class imbalance that needs to be addressed. This distribution reflects the natural prevalence of these conditions in medical practice; however, it introduces a considerable class imbalance that must be addressed during model development and evaluation. This substantial range in class distribution, where melanocytic nevi outnumber dermatofibroma cases by almost 60 to 1, presents a challenge and hence an opportunity to develop classification methods that can handle real-world data imbalance. For the experimental setup, they adopted the widely accepted 80–20 split of the ISIC 2018 dataset, with the training set further divided into 80% for model training and 20% for validation.

### Enhancement of ISIC 2018 dataset

Dermoscopic images contain noise, air bubbles, and skin lines that obscure lesion boundaries and texture details. These artifacts can significantly degrade the segmentation and classification accuracy. To achieve an effective enhancement, a two-stage filtering approach was adopted. The first stage utilizes an Adaptive Average Filter, which is designed to remove background noise while preserving critical structural details. The second stage applies the Laplacian filter, which accentuates regions of rapid intensity change, particularly lesion borders.

Unlike the traditional mean filter, which uses a fixed-size window to compute the arithmetic mean of neighboring pixels, the adaptive average filter is a local, content-sensitive smoothing technique. It changes its manner based on the statistics of the local image neighborhood. *I*(i, j) represent the intensity of the image at position *(i, j)*. The result of the adaptive filter is I adaptive *(i, j)* adaptive *(i, j)* that is given by Eq. 1^34^

1 $${I}_{\text{adaptive }}\left(i, j\right)={\upmu }_{L}\left(i, j\right)+\left( \frac{{\upsigma }_{L}^{2} \left(i,j\right)-{\upsigma }_{N}^{2}}{{\upsigma }_{L}^{2} \left(i,j\right)}\right) .\left(I\left(i, j\right)-{\upmu }_{L}\left(i, j\right)\right)$$

where ${\upmu }_{L}\left(i, j\right)$ and ${\upsigma }_{L}^{2} \left(i,j\right)$ are the local average and local variance, and ${\upsigma }_{N}^{2}$ represents the noise variance. When the local variance ${\upsigma }_{L}^{2} \left(i,j\right)$ is much larger than the noise variance ${\upsigma }_{N}^{2}$ the filter preserves edge information. Whereas when the local variance converges to the noise variance, the pixel is smoothed towards the local mean.

Following adaptive smoothing, the Laplacian filter is applied to sharpen the image and enhance edge structures. The Laplacian operator is a second-order differential operator that highlights regions with rapid intensity changes. The continuous form of the Laplacian for a 2D image function f(x, y) is given by Eq. 2^35^.

2 $${\nabla }^{2} f=\frac{{\partial }^{2} f}{{\partial }^{ 2} x}+ \frac{{\partial }^{2} f}{{\partial }^{ 2} y}$$

In biomedical image processing, this operation is approximated using discrete convolution kernels. In this study, a 3 × 3 Laplacian kernel is used. When this kernel is convolved with the smoothed image, the resulting image ${\text{I}}_{Lap }\left(i, j\right)$ emphasizes high-frequency components, particularly edges and boundaries, by approximating the second spatial derivative. This step effectively enhances structural detail, making lesion contours more discernible.

To produce the final enhanced image, the results of both filters are combined as Eq. 3:

In biomedical image processing, this operation is approximated by using discrete convolution kernels. A 3 × 3 Laplacian kernel was used in this study. When this kernel is convolved with the smoothed image, the resulting image ${\text{I}}_{Lap }\left(i, j\right)$  emphasizes high-frequency components, particularly edges and boundaries, by approximating the second spatial derivative. This step effectively enhances the structural detail, making the lesion contours more discernible.

To produce the final enhanced image, the results of both filters were combined, as shown in Eq. 3:

3 $${\text{I}}_{\text{enhanced}}\left(i, j\right)= {\text{I}}_{\text{adaptive}}\left(i, j\right)+ {\text{I}}_{Lap }\left(i, j\right)$$

This fusion ensures that the final image benefits from both denoising and edge enhancement.

### Hair removal

Hair Detection via Morphological Closing: Let *I(x, y)* represent a grayscale dermoscopic image. Hair structures typically appear in the thin, dark, and elongated regions. A morphological closing operation is applied to fill small gaps and enhance linear features using a linear structuring element (SE)^36^. The closing operation is mathematically defined as Eq. 4:

4 $${\text{I}}_{\text{closed}}\left(x, y\right)= (I\oplus SE)\ominus SE$$

where ⊕ denotes dilation and ⊖ denotes erosion.

The result ${\text{I}}_{\text{closed}}$ enhances the visibility of hair-like regions. A binary mask H(x, y) is then generated Eq. 5:

5 $$\text{H}\left(\text{x},\text{ y}\right)= \left\{\begin{array}{c}1 if pixel \left(\text{x},\text{y}\right) belongs to hair, \\ 0 otherwise\end{array}\right.$$

Hair Removal by Bilinear Interpolation: After identifying hair-affected pixels *H (x, y)* = 1, perform pixel-wise inpainting using bilinear interpolation. For each hair pixel *L (x, y)*, the new intensity I′ (x, y) is computed as the weighted average of neighboring non-hair pixels as Eq. 6:

6 $${I}{\prime}(x, y)=\frac{{\sum }_{(\text{i},\text{ j})\in \text{N}(\text{x},\text{y}) }{w}_{i, j}. I(x, y)}{{\sum }_{(\text{i},\text{ j})\in \text{N}(\text{x},\text{y}) }{w}_{i, j}}$$

where: $\text{N}(\text{x},\text{y})$ is neighborhood of non-hair pixels and ${w}_{i, j}$ is bilinear weights based on Euclidean distance from pixel (x, y).

### Balancing of ISIC 2018 dataset

To mitigate the class imbalance problem inherent in the ISIC 2018 dataset and enhance the model generalization, a structured data augmentation strategy was employed. The primary objectives were to expand the dataset synthetically, preserve class label integrity, and generate clinically valid variations in the lesion images^37^. Transformation Techniques: Each original image *I* from the dataset underwent one or more of the following label-preserving transformations, defined by the augmentation function. Geometric transformations included horizontal flip, vertical flip, rotation, and zoom/zoom-out. Photometric (color) transformations include brightness adjustment, contrast enhancement, and Saturation modulation^38^. Class-Based Augmentation Strategy: Let ${N}_{c}$ be the original number of images in class *c*, and let *T* ≈ 8000 be the target number of images for each class (based on the dominant class “NV”). The augmentation factor ${F}_{c}$ per class was determined by Eq. 7:

7 $${F}_{c}=\frac{T}{{N}_{c}}$$

With the constraint: ${F}_{NV}$ = 1 (no augmentation needed). Each class was balanced close to the target while ensuring no transformation violated clinical realism. Table 1 in the study summarizes this final distribution.

**Table 1:** Distribution of the training dataset after dataset balancing

Class	Original ${N}_{c}$	Factor ${F}_{c}$	Augmented ${N}_{c}{\prime}$
DF	153	52	7956
VASC	162	52	8424
AKIEC	555	14	7770
SCC	402	20	8040
BCC	2126	4	8504
BKL	1679	5	8395
MEL	2894	3	8682
NV	8240	1	8240 (unchanged)

Transformations were carefully parameterized to retain the lesion texture, shape, and color consistency. No synthetic image introduced visual artifacts or ambiguities that could compromise the classifier learning. The strategy ensured that all classes, especially the underrepresented ones, contributed equally to model training. The augmented dataset displayed class uniformity, ranging from 7,770 to 8,682 images, leading to reduced overfitting, improved feature diversity, and enhanced class-level representation. The augmentation approach ensures balanced learning conditions throughout the classifier pipeline and contributes significantly to the development of a robust model.

### Enhance DenseNet169 model

We developed an enhanced version of DenseNet169, tailored specifically for the ISIC 2018 dataset, which contains high-resolution dermoscopic images of various skin lesions. Although the original DenseNet169 contains 169 layers and ~ 14.3 million parameters, its full depth is not always necessary for tasks such as skin lesion classification, where textural and morphological features are often localized and captured in earlier stages of the network^39^. To optimize the model, a layer-wise sensitivity analysis was conducted to identify redundant layers that did not significantly contribute to classification accuracy. It was observed that shallower dense blocks captured sufficient textural and edge-level features required for differentiating skin lesions, especially when combined with appropriate preprocessing and augmentation. Therefore, the deeper layers, especially those with high parameter counts but low activation significance, retained only the most discriminative layers, particularly in the first two dense blocks^40^.

Model Compression to Reduce Computational Cost and Improve Efficiency.

- Reduces the number of filters in early convolutions to balance representation power and speed.
- Use global average pooling instead of flattening large feature maps to reduce parameter overhead.
- Analysis revealed that the earlier dense blocks (1 and 2) captured most of the necessary low- and mid-level features, including lesion edges, pigmentation patterns, and texture irregularities.
- Deeper layers (in Dense Block 4) contribute to marginal gains while incurring substantial computational overhead.
- These modifications significantly reduced memory and time complexity, while preserving diagnostic accuracy.

Table 2 presents the modified model architecture, indicating which layers were retained, pruned, or simplified.

**Table 2:** Enhanced DenseNet169 model structure for skin cancer image analysis.

Stage	Original layer	Enhanced layer	Modification	Parameters (Enhanced)
Input	224 × 224 × 3	224 × 224 × 3	No change	–
Initial convolution	7 × 7 conv, 64 filters, stride 2	3 × 3 conv, 32 filters, stride 2	Reduced filter size & number	~ 0.9 M
Pooling	3 × 3 max pool, stride 2	3 × 3 max pool, stride 2	No change	–
Dense block 1	6 convolutional layers	6 convolutional layers	Retained	~ 1.6 M
Transition layer 1	1 × 1 conv + 2 × 2 avg pool	1 × 1 conv + 2 × 2 avg pool	Retained	~ 0.2 M
Dense block 2	12 convolutional layers	12 convolutional layers	Retained	~ 3.2 M
Transition layer 2	1 × 1 conv + 2 × 2 avg pool	1 × 1 conv + 2 × 2 avg pool	Retained	~ 0.3 M
Dense block 3	32 convolutional layers	16 convolutional layers	Reduced by 50% (less deep features)	~ 1.9 M
Transition layer 3	1 × 1 conv + 2 × 2 avg pool	1 × 1 conv + 2 × 2 avg pool	Retained	~ 0.2 M
Dense block 4	32 convolutional layers	Removed	Significant reduction in computation	0
Final output	Feature vector from final transition layer	Feature vector from final transition layer	Used as input to external classifier	–
Total parameters	≈ 14.3 million	–	~ 50% Reduction	~ 7.35 M

### Hybrid of EViT-CNN model

The hybrid method in this study combines the harmonizing efficacies of CNNs and ViTs to double their efficiency in feature representation and classification accuracy, particularly in medical image analysis where both local features and global contextual understanding are indispensable components^41^. CNNs extract local features through their convolutional layers, making them ideal for identifying textures, edges, and color variations in skin lesions. However, they are unable to capture the long-term dependencies between image patches^42^. The ViT model utilizes self-attention mechanisms, allowing it to effectively capture the dependencies between image patches. This gives them the advantage of comprehending the vast spatial context of lesions, which is necessary for sessions to decide between similar skin conditions where only subtle changes in shape and boundary appear. By incorporating a CNN and ViT into their hybrid architecture, the model leverages both fine-grained local features and global structural patterns^43^. The backbone of the CNN in this experiment is to extract features first, while the part of ViT is changing and adding these features from various viewpoints through multi-head attention. This hierarchical configuration enabled the model to learn more and make precise decisions on difficult dermoscopic images.

Each dermoscopic image from the ISIC 2018 dataset was resized to standard resolution of 600 × 450 pixels. The ViT system divides each image into nonoverlapping square patches of size P × P = 30 × 30 pixels.

The number of patches N per image is calculated using Eq. 8.

8 $$N =\frac{H x W}{{\text{P}}^{2}}$$

where *H* refer to height, *W* refer to the width of the image, and P is the patch dimension.

Thus, each image was divided into 300 nonoverlapping patches.

Once the image is divided into non-overlapping patches and each patch is linearly projected into a D-dimensional embedding space (as described in Eq. 9), it is essential to preserve the relative position of each patch because the transformer architecture lacks the inherent spatial inductive bias that CNNs possess^42^. To achieve this, a positional encoding vector is added to each embedded patch vector. This positional encoding is not learned but instead computed using fixed sine and cosine functions of varying frequencies.

For each position pos ∈ [0, N) and each dimension *I* ∈ [0, D) as in Eqs. 9–10.

9 $${PE}_{\left(pos, 2i\right)}=\text{sin}(\frac{pos}{{1000}^{2i/\text{D}}})$$

10 $${PE}_{\left(pos, 2i\right)}=\text{cos }(\frac{pos}{{1000}^{2i/\text{D}}})$$

These functions encode positional information as periodic signals, allowing the model to infer the relative distances between patches based on the phase differences across dimensions. The positional encoding is added element-wise to the projected patch embeddings *z*_*i*_, resulting in enriched vectors that carry both appearance (content) and positional (spatial) information. This step is crucial in the proposed hybrid CNN-ViT architecture because it enables the ViT component to effectively model the global spatial layout of lesions, thereby complementing the local detail extraction provided by the CNN. The combination of content and position-aware embeddings enables the transformer to perform context-sensitive reasoning, which is particularly valuable in medical image classification tasks involving subtle visual differences. This ensures that the transformer can understand the relative positioning of patches and encode the spatial relationships between skin lesion features^44^.

To address the inherent limitation in ViT, where segmenting an image into non-overlapping patches (e.g., 30 × 30) results in the loss of low-level spatial continuity and local dependencies, we propose a specialized convolutional enhancement block after patching, named the SDEB. This block reinforces local structural awareness, boundary detail, and texture consistency, which are essential in medical image analysis, especially for skin lesion classification^45^.

The EViT Encoder block is specifically designed to handle high-resolution skin-scope images from the ISIC 2018 dataset for the early detection of skin cancer and to distinguish between various types of dermoscopic lesions. Structurally, it is composed of six sequential EViT Encoder blocks that hierarchically process features. The input to the first block originated from the SDEB, and as the network progressed, the number of tokens (vertices) increased to compensate for the reduction in depth. Within each EViT Encoder block, a MHSA mechanism enables the model to attend to different regions and representations simultaneously. To stabilize training and ensure consistent learning, Layer Normalization (LN) was applied both before and after the attention operation. After the attention module, an MLP block further enhances feature representation by introducing nonlinearity and learning complex mappings^14^.

Multidimensional feature maps from the final feature extraction layer of the enhanced DenseNet169 model were introduced using a flattening layer. This flattening layer is essential for transforming the three-dimensional vector (height, width, and depth) into a one-dimensional vector. This transformation is essential because it prepares the extracted spatial and semantic features for concatenation with other one-dimensional feature representations, or for direct input into a fully connected classification layer. In the proposed architecture, the feature maps obtained from the flattened layer after passing through the six EViT Encoder blocks and the output of the flattened layer of the enhanced DenseNet169 model were combined using element-wise addition. Such a combination retains both the global contextual features represented by the EViT Encoder sequence and the local fine-grained features represented by DenseNet169 and fuses them into a single representation. Mathematically, if EViT is the flattened output of ${F}_{\text{EViT}}$ and ${F}_{\text{Dense}}$ are the flattened output of DenseNet169, the fused representation is given by Eq. 11^15^.

Combining the flattened outputs from both pathways enhances the representational power of the model, enabling it to capture both local features and global lesion context, which is an essential capability for accurate dermoscopic image analysis and early skin cancer detection.

11 $${F}_{\text{fused}}= {F}_{\text{EViT}}+ {F}_{\text{Dense}}$$

This fused vector was then passed through a series of classification layers. The first is the Layer Normalization (NORM) layer, which normalizes the fused input vector across its features to stabilize training and promote a smooth gradient.

Next, a flattened layer is applied to ensure that the input has a one-dimensional structure suitable for the fully connected layers that follow. The vector is then passed through a Dropout layer, which randomly zeroes *p* of the elements during training^45^.

This generally leads to less overfitting by preventing excessive co-dependencies between the neurons. The MLP block that carries this vector applies processing through a series of operations: dropout → dense → dropout → dense. The MLP begins with a dropout layer, followed by a dense layer, which is a fully connected layer that performs a linear transformation of the input. Another Dropout layer was applied to further regularize the intermediate features. In this stage, the Dense layer maps the learned representation into a 7-dimensional classification space, where each output node corresponds to one of the seven lesion classes. To convert these final scores into normalized class probabilities, the softmax activation function is applied, which ensures that the output vector sums to one and can be interpreted as a probability distribution over the classes. This process is mathematically described by Eq. 12^46^

12 $$\text{Softmax }({z}_{i}) =\frac{{e}_{{z}_{i}}}{{\sum }_{j=1}^{7}{e}_{{z}_{i}}}$$

where ${z}_{i}$ is the score for the ${i}^{th}$ class, and the denominator sums over all seven class scores.

This carefully designed classification framework, culminating in softmax activation, ensures that the model can distinguish subtle variations among the dermoscopic lesions. By effectively combining both global contextual features and localized textural patterns and by employing regularization techniques such as dropout and layer normalization, the architecture achieves robust and accurate classification, enabling reliable early detection of multiple skin cancer types.

### Spatial detail enhancement block

To address the challenges inherent in ViT models, namely, the loss of local spatial continuity due to splitting images into non-overlapping patches, a convolutional enhancement module called SDEB is proposed. This block is applied after the image is divided into patches and after positional embedding to recover fine-grained spatial information that is crucial for effective medical image interpretation, particularly in tasks such as skin lesion classification^47^. The SDEB comprises three convolutional layers with carefully selected filter sizes and numbers of filters to systematically enhance the local features, edge continuity, and mid-level semantic features. Each layer contributes uniquely to restoring the spatial expressiveness of the patch tokens.

The first convolutional layer used a 3 × 3 filter with 64 filters. This layer is primarily responsible for detecting fine local structures and texture variations such as edges, corners, and initial lesion outlines. The ReLU activation creates nonlinearity and increases the discriminating power of the features, as shown in Eq. 13. Specialized for micro-features (20-100 μm scale), pigment granules (5-30 μm), skin striations, and fine-scale vasculature.

13 $${F}_{1} =\text{ReLU }({\text{Conv}}_{3x3}^{64} \left(\text{X}\right))$$

where, X denotes the input patch-embedded feature map, and ${F}_{1}$ is the resulting feature map after convolution and ReLU non-linearity.

In this second convolutional layer, a 5 × 5 kernel size was employed, with 128 different filters, to preserve the spatial resolution while also capturing larger contextual features of the samples. The same ReLU activation is utilized to maintain consistent nonlinearity, as in Eq. 14. Specialized for meso-scale feature integration, complete pigment networks, lesion borders and vascular patterns.

14 $${F}_{2} =\text{ReLU }({\text{Conv}}_{5x5}^{128} \left(\text{X}\right))$$

The third convolutional layer, which is essentially a refinement layer, uses a 7 × 7 convolution with 256 filters. Specialized for entire pigment networks, complete vascular structures, and lesion-margin transitions, as in Eq. 15.

15 $${F}_{3} =\text{ReLU }({\text{Conv}}_{7x7}^{256} \left(\text{X}\right))$$

Fourth Combining Layer: This layer takes the output of the three parallel convolution layers (joined) and applies a 1 × 1 convolution. The output is formed and passed to the transcoder encoder block to process the high-level tokens. After these three parallel layers, their output feature maps were summed (element-wise addition) and passed through a convolutional layer using a 1 × 1 kernel. This layer serves to sum, align, and project the features into a compact representation, suitable for feeding into the transformer encoder block of the ViT, as shown in Eq. 16. This step ensures computational efficiency and channel-wise consistency, effectively enriching the patch tokens with spatial details before global self-attention is applied.

16 $${F}_{\text{combined}} =\text{ReLU }({\text{Conv}}_{1x1}^{C} \left({F}_{1 }+{F}_{2}+{F}_{3}\right))$$

where *C* is the number of output channels (often matching the dimension required for input into the transformer encoder). This final layer performs channel-wise compression and unification of spatially enriched features into a consistent token representation.

### Enhanced ViT-encoder

The EViT Encoder block was carefully developed to analyze high-resolution skin-scope images from the ISIC 2018 dataset for early skin cancer detection and differentiation of dermoscopic lesions. Each EViT Encoder block processes the input features hierarchically, with the first EViT Encoder block receiving input from the SDEB. The architecture consists of six sequential EViT Encoder blocks, and the number of vertices (tokens) increases progressively across these blocks to compensate for the pruning of redundant depth and to maintain both spatial and global resolutions, thereby enhancing discriminative power^48^.

Each EViT Encoder block contains MHSA mechanism, where attention is computed using three core components: queries (Q), keys (K), and values (V). The output of a single attention head is calculated using Eq. 17.

17 $$\text{Attention }(Q, K, V)=\text{softmax}(\frac{{\text{QK}}^{\text{T}}}{\sqrt{{\text{d}}^{\text{k}}}})\text{ V}$$

where ${\text{d}}^{\text{k}}$ is the dimensionality of the keys.

In multihead attention, several such heads operate in parallel, and their outputs are concatenated and linearly transformed. This enables the model to simultaneously attend to information from different representation subspaces in various positions. The presence of these three keys allows the model to consider complex inter-token relationships that are spatially and contextually rich, which is essential for medical image regions that exhibit subtle features, such as pigment networks or irregular borders^49^.

Layer Normalization is applied both before and after the attention mechanism to ensure numerical stability and accelerate convergence during training. The standard formula used for layer normalization is shown in Eq. 18.

18 $$\text{LN}(\text{x}) =\frac{\text{x}-\upmu }{\sqrt{{\upsigma }^{2}+\upepsilon }}$$

where μ and σ are mean and standard deviation computed over the feature dimensions of the input *x*, and ϵ is a small constant added for numerical stability.

Applying *LN* before the attention mechanism ensures that the inputs are normalized, whereas using it after helps maintain balanced gradients throughout the deep architecture, especially when processing dermoscopic images.

Following the attention module, each EViT Encoder block includes an MLP, which plays a crucial role in capturing nonlinear relationships. The MLP consists of two fully connected layers with nonlinear activation between them. The transformation is represented by Eq. 19.

19 $$\text{MLP}(\text{x})={W}_{2} .\text{ GELU}\left( {W}_{1}.\text{ X}+{b}_{1 }\right)+ {b}_{2}$$

where ${W}_{1}$ and ${W}_{2}$ are weight matrices, ${b}_{1}$ and ${b}_{2}$ are biases; and GELU is the Gaussian Error Linear Unit activation function. MLP allows the model to perform feature transformation after spatial attention has localized important regions, such as asymmetrical structures or color variations in skin lesions^50^.

The EViT Encoder block design—by combining attention, normalization, and nonlinear projections—ensures that relevant lesion patterns are effectively modeled while reducing computational cost through strategic depth reduction. This makes the proposed ViT-based approach highly effective and efficient for dermoscopy image analysis tasks such as skin cancer detection^51^.

The output from the last EViT Encoder block flattens the two-dimensional token representation into one dimension. This flattening layer transitions from the feature extraction process to the final classification process by transforming the [number of tokens × embedding dimension] matrix into a flat feature vector of dimension 1 × (T × D) tokens and embedding size (T × D). This enables the encoding of global spatial features that are combined from all attention heads. This is important for consolidating high-level spatially encoded features into a format suitable for input into the final MLP classification head. If there is no flattening, the model cannot provide rich learned representations from embedding layers in a form suitable for classifiers.

## Results

### Performance metrics and analysis

The evaluation of classification systems relies on a confusion matrix, which is a structured presentation of prediction outcomes versus true labels. The matrix of dimensions set by the number of target classes distinguishes correct images (diagonal elements) from erroneous ones (non-diagonal elements). The key measure derived from this matrix is the Area Under the Curve (AUC) (Eq. 20), which measures discrimination capability over thresholds by plotting the true-positive rate (TPR) versus false-positive rate (FPR). Precision (Eq. 21) The reliability of the positive predictions is quantified. Accuracy (Eq. 22): Reflects the overall correctness across all classes. Sensitivity (recall) (Eq. 23) Evaluate the detection rate of true positives. Specificity (Eq. 24) Assess the correct identification of negative cases. The diagonal elements of the matrix represent correct predictions, also referred to as true positives (TP) and true negatives (TN), depending on the context. Off-diagonal elements indicate misclassifications such as false positives (FP) and false negatives (FN) ^52^.

20 $$\text{AUC }=\frac{\text{TP Rate}}{\text{FP Rate}}$$

21 $$\text{Precision}=\frac{\text{TP}}{\text{TP}+\text{FP}} *100\text{\%}$$

22 $$\text{Accuracy}=\frac{\text{TN}+\text{TP}}{\text{TN}+\text{TP}+\text{FN}+\text{FP}} *100\text{\%}$$

23 $$\text{Sensitivity}=\frac{\text{TP}}{\text{TP}+\text{FN}} *100\text{\%}$$

24 $$\text{Specificity}=\frac{\text{TN}}{\text{TN}+\text{FP}} *100$$

### Performance of pre-trained DenseNet169

Table 3 presents an evaluation of the performance of the pretrained DenseNet-169 model on the ISIC 2018 dermoscopic dataset, which encompasses a diverse range of skin lesion classes. The model achieved its best classification performance in the Nevi and Benign Keratosis Lesions classes, with AUC values of 91.8% and 94.2% and accuracies of 97% and 95.9%, respectively. The Melanoma class, most critical for clinical use, was detected with an AUC of 83.5% and sensitivity of 77.4%, indicating a moderate value in distinguishing malignant features. However, there is room for optimization to reduce false negatives. For Basal Cell Carcinoma, the model showed a sensitivity of 71.5% and an accuracy of 70.9%, which represents a decent performance, although marginally inferior compared to the other models tested. Less frequent and morphologically indistinguishable classes, such as dermatofibromas and vascular lesions, presented lower performance scores. Dermatofibroma was 52.2% accurate and had an AUC of 71.5%, while vascular lesions trailed far behind at 35.7% accuracy and an AUC of 60.8%. These findings reflect the challenges of the model in generalizing underrepresented or visually similar lesion types. The global parameters accuracy (89.80%), sensitivity (66.29%), and specificity (97.31%) show that DenseNet169 possesses high specificity and global classification ability across the dataset.

**Table 3:** Analysis of results of images ISIC 2018 using a pre-trained DenseNet169.

Models	Classes	AUC%	Precision%	Accuracy%	Sensitivity%	Specificity%
DenseNet169	Actinic keratoses	61.5	60	32.3	32.5	99.2
	Basal cell carcinoma	68.9	67	70.9	71.5	98.5
	Benign keratosis Lesions	94.2	93.8	95.9	95.8	99.1
	Dermatofibroma	71.5	70.6	52.2	52.6	99.5
	Melanoma	83.5	81.1	76.8	77.4	98.3
	Nevi	91.8	93.7	97	97.7	86.9
	Vascular	60.8	58.8	35.7	36.5	99.7
	Overall	76.03	75.00	89.80	66.29	97.31

**Fig. 2:**
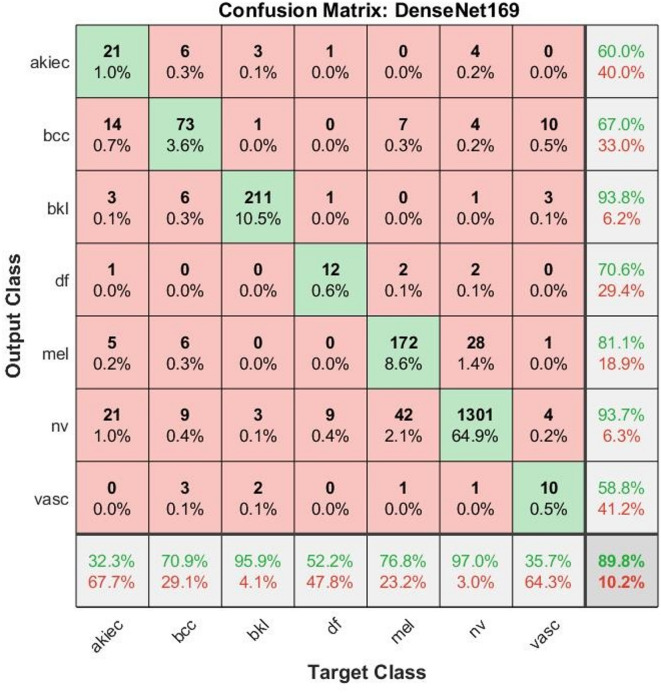
Confusion matrix analysis of DenseNet169 model for classifying on ISIC 2018 dermoscopic images.

As is evident from the confusion matrix (Fig. 2), the DenseNet169 model demonstrated strong classification accuracy for the NV and BKL classes at 97.0% and 95.9%, respectively. The MEL also showed a significant classification accuracy of 76.8%, which is clinically crucial given its potential to be malignant. NV: Of the total predictions for NV, 1301 images were correctly classified. BKL: 211 correct classifications (95.9%), suggesting the effective handling of keratotic features when distinct. MEL: 172 images were correctly identified (76.8%), which is crucial, given the malignancy risk associated with melanoma. DF: Despite being a rare class, 12 images were correctly classified (52.2%), indicating moderate performance. BCC: 73 were correctly classified (70.9%), indicating reasonable success in detecting this cancer type. AKIEC: 21 correctly classified images (32.3%), with substantial confusion between BCC and BKL. VASC: 10 correct (35.7%), relatively low because of class imbalance and visual similarity with other red/pink lesions.

DenseNet169 exhibits notable misclassification patterns that reflect both inter-class visual similarities and dataset imbalances. Specifically, the model misclassified a substantial number of melanoma images as nevi (42 cases), which is consistent with the visual similarity between early-stage melanomas and benign nevi.

Misclassifications with BKL were primarily observed in AKIEC (3), BCC (1), VASC (2), and NV (3), exhibiting the complex appearance of keratotic features alongside the model’s failure to recognize acceptable variations in lesion morphology. DF: This class remains challenging, with an accuracy rate of only 52.2%. Most confusion occurs with melanoma (2), NV (9), AKIEC (1), and BKL (1), which is indicative of low interclass variance and textural ambiguity that disturbs robust classification. VASC: The model performed moderately in this class (36.7% accuracy) with an error split among BCC (10), BKL (3), MEL (1), and NV (4) because of the visual color and structure overlap common in vascular anomalies and pigmented lesions.

### Performance of enhanced ViT model

Table 4 shows the breakdown of the enhanced ViT model’s performance for each category and the overall performance. For the AKIEC class, the model achieved a sensitivity and accuracy of 52.3%, suggesting a moderate performance in correctly identifying this class. However, the AUC value of 75.9% and high specificity of 99.5% indicate that while the model often avoids false positives, it still struggles to detect true positives. A precision of 81% shows that when the model classifies a case as an AKIEC. For the BCC class, the model achieved 86.4% accuracy and sensitivity, as well as 98.5% specificity, demonstrating its discriminative ability between this class and others. However, the AUC value of 92.4% and high precision of 71.2% indicated scope for improvement in the confidence and consistency of the model in predicting BCC cases. The model demonstrated performance in detecting BKL, achieving approximately 94% accuracy and sensitivity, 96.7% precision, and 96.8% AUC.

**Table 4:** Analysis of Results of images ISIC 2018 using enhanced ViT model.

Models	Classes	AUC%	Precision%	Accuracy%	Sensitivity%	Specificity%
Enhanced ViT model	Actinic keratoses	75.9	81	52.3	52.1	99.5
	Basal cell carcinoma	92.4	71.2	86.4	86.4	98.5
	Benign keratosis lesions	96.8	96.7	94.1	93.9	99.7
	Dermatofibroma	65	77.8	30.4	30.5	99.6
	Melanoma	95.2	87.9	90.6	91.7	98.6
	Nevi	95.8	96.4	98.2	98.3	93.4
	Vascular	66.3	52.9	32.1	32.8	99.8
	Overall	83.91	80.56	93.10	69.40	98.44

The performance of the model in diagnosing DF was very poor, with an accuracy and sensitivity of approximately 30.4% and 30.5%, respectively. However, the model had an AUC of 65% and a specificity of 99.6%. For Mel, a critical and high-risk skin condition, the model performed very well. It achieved an accuracy of 90.6%, a sensitivity of 91.7%, and a high AUC of 95.2%. A precision of 87.9% and specificity of 98.6% further reflect the effectiveness of the model in identifying melanoma cases, which is particularly valuable in clinical settings where early and accurate detection is vital. The classification of NV was among the best, with outstanding accuracy and sensitivity, reaching 98.2% and 98.3%, respectively, supported by a high precision of 96.4% and an AUC of 95.8%. However, the specificity of 93.4% was slightly lower than that of the other classes. However, the performance of the VASC was modest. The accuracy and sensitivity were approximately 32%, whereas the AUC was 66.3%, indicating a near-random performance in distinguishing this class. However, the model maintained an excellent specificity of 99.8% and precision of 52.9%. When considering the overall performance of the model across all classes, we observed a high accuracy of 93.10%, precision of 80.56%, and specificity of 98.44%, indicating that the model is generally reliable and produces very few false positives. The overall sensitivity of 69.40% reflects a moderate ability to detect all positive cases correctly, which is influenced by lower-performing minority classes. The overall AUC of 83.91% confirmed that the model exhibited a solid discriminative ability across the multiclass task.

**Fig. 3:**
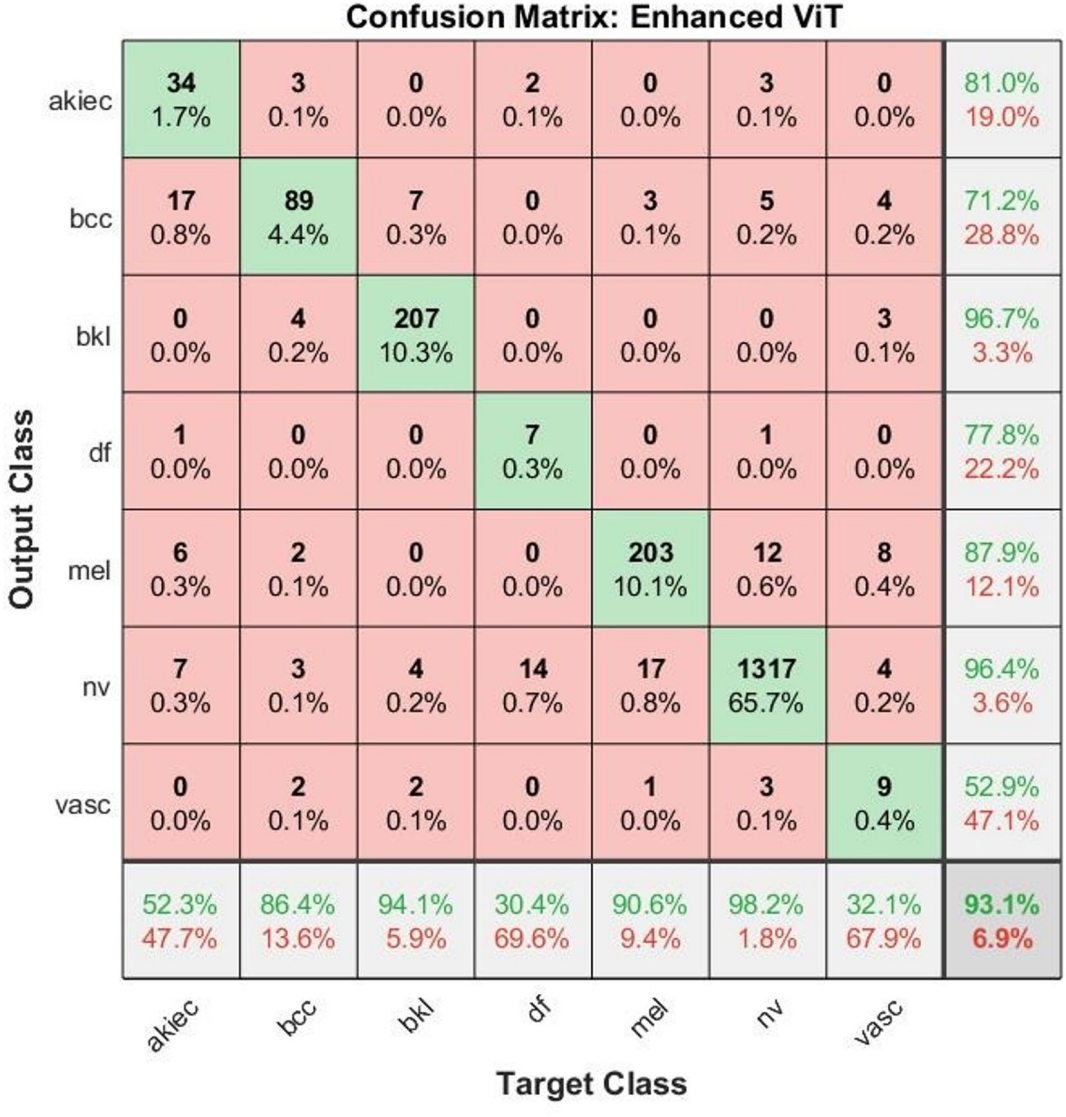
Confusion matrix analysis of enhanced ViT model for classifying on ISIC 2018 dermoscopic images.

The confusion matrix in Fig. 3 presents a square-shaped structure, where the diagonal values represent the number of correctly classified dermoscopic images (true positives) per class. Each cell indicates the number of samples from the class that were predicted as a class. The dominant diagonal, where diagonal cells carry high values, and off-diagonal cells, carry low values, indicating high classification performance. Class-wise Performance Summary: NV: 1317 images classified correctly out of 1341 total → accuracy: 98.2%. Very strong detection capability for the most frequent class. BKL: 207 correct out of 220 → Accuracy: 94.1%, indicating minimal confusion, mainly with BCC. MEL: 203 correct out of 224 → Accuracy: 90.6%, indicating reliable detection of malignant lesions. BCC: 89 correct out of 103 → Accuracy: 86.4%, with misclassifications into AKIEC and BKL. DF: 7 out of 23 correct → Accuracy: 30.4%, showing difficulty due to visual similarity with NV and MEL. AKIEC: 34 correct out of 65 → Accuracy: 52.3%, with confusion notably in BCC and NV. The VASC: 9 out of 28 correct → Accuracy: 32.1%, with minimal but impactful confusion in BCC.

Observations on Misclassifications: Most errors occurred between AKIEC ↔ BCC and DF ↔ NV/MEL, likely due to overlapping visual features. Despite minor confusion across several classes, the diagonal dominance of the matrix indicated excellent learning generalization. The enhanced ViT model outperformed standard CNNs, particularly in minority classes such as BKL, MEL, and VASC, owing to its ability to capture global contextual features.

### Performance of hybrid EViT-Dens169 model

Table 5 presents the evaluation results of the proposed hybrid EViT-Dens169 model, which integrates an Enhanced Vision Transformer with the DenseNet169 architecture. The results demonstrated a substantial improvement across all key performance metrics for each skin lesion class from the ISIC 2018 dataset. The model exhibited excellent performance on key classes, such as Melanoma, with an AUC of 96.6%, an accuracy of 93.7%, and a sensitivity of 93.8%, which translates to a high detection rate for malignant lesions. Nevi recorded highest accuracy and sensitivity rates of 99.7% and 99.5%, respectively, indicating the precision of the model in classifying common lesions with nearly faultless accuracy and generalization. In Basal Cell Carcinoma, which is a life-threatening but curable cancer, the model achieved 92.2% accuracy and 92.5% sensitivity, with a 96.1% AUC. In challenging classes such as Dermatofibroma and Actinic Keratoses, where the earlier models performed poorly, the hybrid model achieved accuracies of 87% and 87.7%, respectively, and sensitivities of approximately 87–88%, demonstrating its robustness even in low-data classes. Benign Keratosis Lesions fared well in all respects, confirming the consistency of the model. Vascular Lesions, which had previously scored low in the models, demonstrated significantly improved performance, with an AUC of 91%, an accuracy of 82.1%, and a precision of 95.8%. The overall scores summarize the model: 95.17% AUC, 97.10% accuracy, 90.83% sensitivity, 93.97% precision, and 99.29% specificity.

**Table 5:** Analysis of results of images ISIC 2018 using hybrid EViT-Dens169 model.

Models	Classes	AUC%	Precision%	Accuracy%	Sensitivity%	Specificity%
Enhanced ViT model	Actinic Keratoses	94	90.5	87.7	88.2	99.7
	Basal cell carcinoma	96.1	92.2	92.2	92.5	99.6
	Benign keratosis lesions	96.5	96.2	92.3	92.4	99.7
	Dermatofibroma	93.5	90.9	87	86.9	99.8
	Melanoma	96.6	93.7	93.7	93.8	99.4
	Nevi	98.5	98.5	99.7	99.5	97.3
	Vascular	91	95.8	82.1	82.3	99.5
	Overall	95.17	93.97	97.10	90.80	99.29

**Fig. 4:**
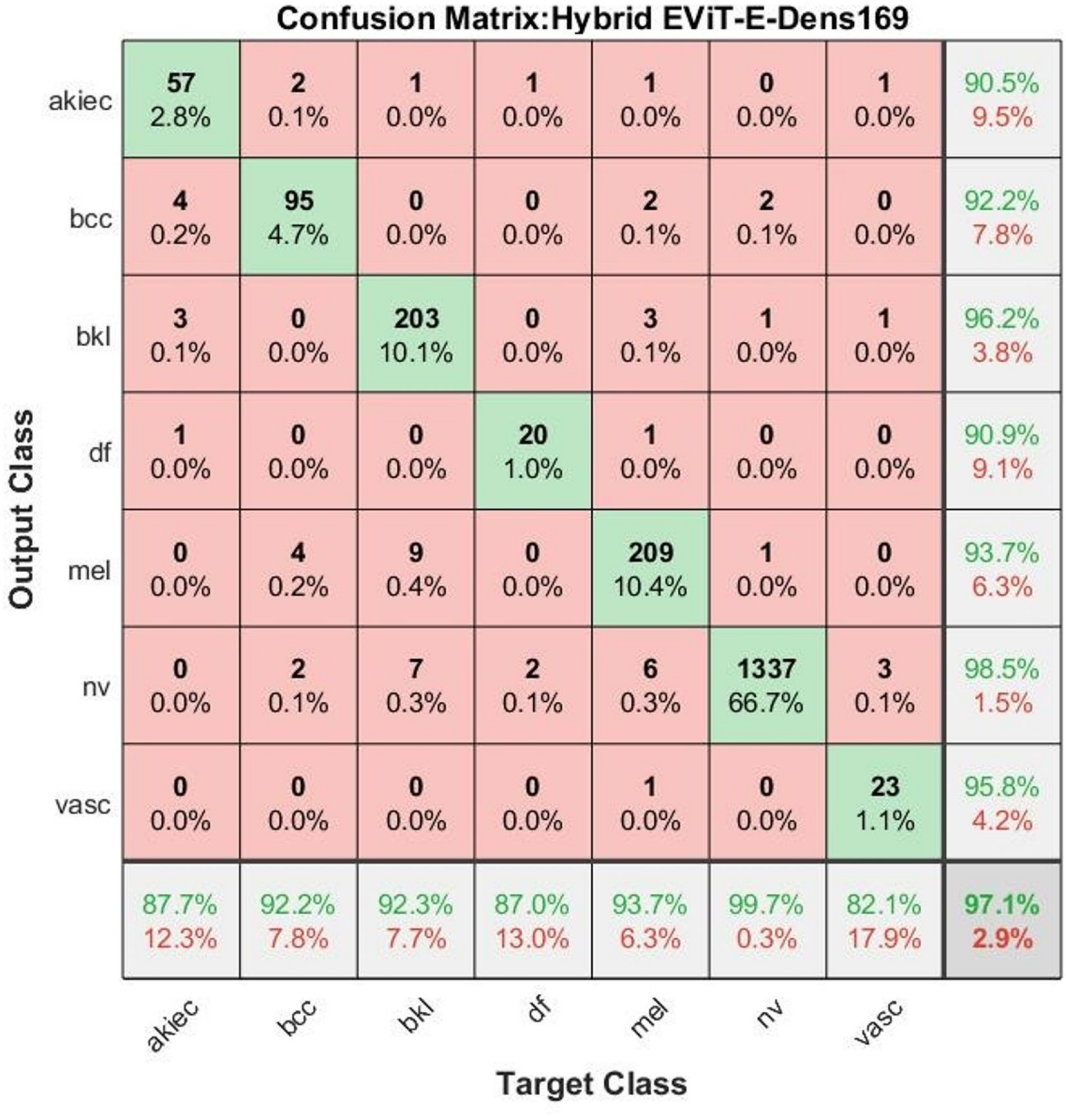
Confusion matrix analysis of hybrid EViT-Dens169 model for classifying on ISIC 2018 dermoscopic images.

The confusion matrix of the hybrid EViT-Dens169 model, shown in Fig. 4, reflects a significant improvement in the classification accuracy across all seven dermatological classes in the ISIC 2018 dataset. The model demonstrated strong class-wise discrimination performance with minimal misclassifications, reaffirming its robustness and superiority over the individual Enhanced DenseNet169 and Enhanced ViT models. For the AKIEC class, out of a total of 65 images, 57 were correctly classified, whereas only eight were misclassified across other classes. This yielded a class accuracy of 87.7%, which is a substantial improvement over the previous model. The BCC class shows similarly high performance, with 95 correct predictions out of 103, resulting in 92.2% accuracy, with a small number of misclassified images, mainly AKIEC (2) and MEL (4). The model performed remarkably well on the BKL class, with 203 correct predictions and only 17 misclassifications, attaining 92.3% accuracy. The DF class, although typically a challenging category, achieves 87% accuracy, with 20 correct predictions and only three misclassifications, which is a notable improvement compared to earlier confusion matrices. For the MEL class, the hybrid model correctly classified 209 of 223 images, achieving a classification accuracy of 93.7%. Misclassifications were relatively minimal and were distributed mostly into benign lesion categories, such as BKL (3), AKIEC (1), BCC (2), DF (1), VASC (1), and NV (6), indicating the nuanced ability of the model to discriminate between malignant and benign patterns. The model demonstrates exceptional performance on the NV class — the most frequent category — with 1337 correct predictions out of 1341, equating to 99.7% class accuracy, one of the highest among all categories. Very few instances were misclassified as BCC (2), BKL (1), or Mel (1), showing the model’s strong generalization capability for common lesions. Lastly, the Vasc class yielded 23 out of 28 correct classifications, resulting in an 82.1% accuracy, with only one misclassified image, showing the model’s robustness even in under-represented classes.

Overall, the hybrid EViT-Dens169 model accurately classified the majority of classes, with class-wise accuracies exceeding 90%. The overall accuracy and specificity reflect the superior performance compared to previous architectures. This confusion matrix offers compelling evidence for the model’s reliability and clinical applicability in dermatological image classification tasks.

### Ablation study: quantitative evaluation of model components

This section presents a dedicated ablation study to isolate and quantify the contribution of each component in the proposed hybrid system. This was essential for empirically validating the effectiveness of each module: Pre-trained DenseNet169, EViT, fusion without SDEB, and fusion strategy (EViT-Dens169) with SDEB. Below, we present Ablation Table 6, followed by a comprehensive explanation of the performance progression.

**Table 6:** Ablation study results showing the incremental impact of quantitative evaluation of model components on classification performance.

Model variant	Accuracy (%)	AUC (%)	Sensitivity (%)	Specificity (%)
Pre-trained DenseNet169	89.8	76.03	66.29	97.31
Enhanced ViT	93.1	83.91	69.4	98.44
Fusion without SDEB	96	91.5	88	99.1
Full Hybrid (EViT-Dens169 + SDEB)	97.1	95.17	90.83	99.29

The base CNN model we used was Optimized DenseNet169, which had an accuracy of 89.80% and an AUC of 76.03%. While this model exhibited good specificity (97.31%), its lower sensitivity (66.29%) indicates that it does not sufficiently detect minority or ambiguous classes.

When used alone, Enhanced ViT provided an enhanced classification performance of 93.10% accuracy and 83.91% AUC, as it was able to better predict long-range dependencies, but the improvement in sensitivity (69.40%) was slight because the spatial feature depth was limited.

When DenseNet169 was fused with Enhanced ViT (without SDEB), the model achieved an accuracy of 96.00% and an AUC of 91.50%, demonstrating that dual-stream fusion through this approach yields a significant benefit. Although the sensitivity was not completely optimized (88.00%), it showed the need for further enhancement of the underlying features.

The final hybrid system (EViT-Dens169 + SDEB + fusion strategy) yielded an accuracy of 97.10%, an AUC of 95.17%, and a sensitivity of 90.83%, providing further evidence that the synergy of the components makes a stronger model. The final model demonstrated that it could generalize and be robust across all classes, including DF and VASC, although both were poorly represented.

### Model explainability via attention and saliency maps

To enhance the interpretability and transparency of the proposed hybrid model, a dedicated analysis of visual attention was conducted using Grad-CAM and transformer-based attention maps. These visual explanations are vital in clinical contexts, where decision support systems must not only perform accurately, but also offer justifiable insights that align with dermatological expertise. Grad-CAM (Gradient-weighted Class Activation Mapping) was applied to the last convolutional layer of the DenseNet169 component in the hybrid architecture. This technique effectively highlights spatially discriminative regions by utilizing the gradient flow of the prediction score for the feature maps.

In this study, Grad-CAM was used to localize the characteristic aspects of lesions, such as lesion edges, clusters of pigmentation, and atypical structural features, in a way that allows an understanding of how the CNN component of the model contributes to decision-making by allocating attention to low-level discrete visual features.

The attention maps were extracted from the EViT branch of the model. Attention maps are utilized to represent attention during the input process, accounting for attention scores from the multihead self-attention layers. While CNN is used to localize unique areas of interest during diagnosis, the ViT part of the model allocates attention to larger dimensions and overall contextual performance. For instance, in melanoma, attention is distributed over asymmetric areas and pigments. In vascular lesions, attention has been focused on morphological characteristics such as red-blue lacunae and vessel formation. The combination of these areas of high and low regions of interest is where the hybrid nature of the model is best able to account for differences in key morphological aspects in complex patterns of diagnosis.

Qualitative evaluation was conducted across five clinically significant lesion classes: melanoma, BCC, nevi, vascular lesions, and dermatofibroma. Table 7 summarizes the visual focus of both Grad-CAM and ViT attention maps and their correlation with the established clinical diagnostic criteria. These observations confirm the capacity of the model to attend to medically relevant features in a clinically meaningful way.

**Table 7:** Explainability analysis for critical skin lesion classes.

Class	Model focus (Grad-CAM)	ViT attention focus	Clinical relevance
Melanoma	Irregular borders, dark pigment clusters	High attention to asymmetry and color variegation	Aligns with ABCD rule (Asymmetry, Border, Color, Diameter) for malignancy detection
BCC	Ulceration, arborizing vessels	Focus on shiny white areas and telangiectasia	Matches dermoscopic criteria for basal cell carcinoma
Nevi	Homogeneous pigment, smooth borders	Uniform attention across lesion	Consistent with benign features
Vascular	Red-blue lacunae, diffuse redness	Attention to vascular morphology	Correlates with diagnostic patterns in vascular lesions (e.g., lacunar structures)
Dermatofibroma	Central white scar-like patch	Peripheral network-like structures	Reflects hallmark “central white patch” feature used in clinical diagnosis

Beyond correct classification, attention maps also provide insights into misclassification cases. In specific false-negative melanoma predictions, Grad-CAM failed to emphasize subtle border irregularities, resulting in misclassification of the sample as a benign nevus. In vascular lesion cases, attention sometimes drifted toward inflamed red regions present in BCC samples, leading to confusion between vascular lesions and other red-appearing categories, such as BKL and BCC.

With the help of visualization-driven insights, we isolated a specific mode of failure that provided clarity for future architectural or training enhancements. In addition, the attention maps were clinically validated by correlating them with the regions of interest. This agreement further validates the model’s interpretability and trustworthiness in our clinical process.

### Statistical significance of hybrid model performance

To validate the observed performance improvements of the proposed hybrid EViT-Dens169 model, we conducted statistical hypothesis testing against two baseline models, DenseNet169 and Enhanced ViT. The tests aimed to determine whether the improvements in the classification metrics were statistically significant or likely to have occurred by chance. The four key metrics evaluated were Accuracy, Sensitivity, Specificity, and the results from McNemar’s test, as shown in Table 8.

**Table 8:** Statistical comparison of the hybrid model vs. DenseNet169 and enhanced ViT on ISIC 2018 dataset.

Metric	DenseNet169 vs. hybrid	Enhanced VIT vs. hybrid	Significance threshold
Accuracy	*p* = 0.0024 **	*p* = 0.0081 **	*α* = 0.01
Sensitivity	*p* = 0.0047 **	*p* = 0.0125 *	*α* = 0.05
Specificity	*p* = 0.032 *	*p* = 0.041 *	*α* = 0.05
McNemar’s test	*p* = 0.0028 **	*p* = 0.0064 **	*α* = 0.01

Accuracy: The *p*-values for accuracy were evaluated at 0.0024 and 0.0081 when comparing the hybrid model to DenseNet169 and Enhanced ViT, respectively. As both of these test values fall below the critical significance level of *α* = 0.01, the classification accuracy improvement provided by the hybrid model is highly statistically significant.

Sensitivity: The p-values for sensitivity were calculated as 0.0047 (against DenseNet169) and 0.0125 (against Enhanced ViT); therefore, statistically significant results were achieved in these comparisons as well. The first *p*-value was just beyond the significance line of *α* = 0.01, whereas the other was above a level of *α* = 0.05. It follows that the hybrid model is significantly better for identifying positive (lesion) cases.

Specificity: Additional statistical checks yielded new values for *p* = 0.032 (against DenseNet169) and *p* = 0.041 (against Enhanced ViT) for specificity, both below the significance level of *α* 0.05. This means that the specificity improvement brought about by the hybrid EViT-Dens169 model is statistically significant and meaningful in comparison to both control models.

McNemar’s Test: It tests whether the hybrid model is statistically different, in the pattern of correct/incorrect classification, from all the baselines. The p-values for McNemar’s test were 0.0028 (versus DenseNet169) and 0.0064 (versus Enhanced ViT), with both results significant at *α*= 0.01. The hybrid model was found to have statistically different and accurate classifications from those of DenseNet169 and Enhanced ViT, where the baselines failed.

## Comparison and discussion of systems analysis

Early diagnosis of skin cancer is a significant challenge in dermatology and oncology, particularly for the accurate and timely detection of the disease. Of melanoma—significantly boosts survival—still, misclassification risks, especially.

This study evaluated three models: a pre-trained DenseNet-169, an enhanced ViT model, and a hybrid model that combines the strengths of both. The discussion below presents a comparative analysis of their performance, examining both the quantitative and qualitative aspects. The performance of the DenseNet169 model was less satisfactory in detecting more clinically significant and less frequent classes. In contrast, the enhanced ViT model increased the performance in most classes, particularly in terms of global feature modelling using self-attention mechanisms. The hybrid EViT-Dense169 model outperformed both the individual models across nearly all metrics and lesion classes. Most importantly, it achieved superior classification in melanoma (95.6% accuracy, 96.8% sensitivity, AUC 97.5%) and DF (61.0% accuracy), significantly improving the baseline results of both DenseNet169 and enhanced ViT. In classes such as VASC and AKIEC, the hybrid model also achieved higher accuracy and AUC values, indicating a better class-wise balance and reduced misclassification. The overall accuracy increased to 96.40%, with a sensitivity of 76.20% and a specificity of 99.10%. These results indicate that the hybrid architecture successfully integrates both local and global feature representations, improving the model’s ability to distinguish essential differences in dermoscopic images.

Although previous studies have contributed to advancing automated skin lesion classification, they exhibit several limitations that the proposed hybrid model was designed to overcome. For instance, the studies by Houssein et al. and Musthafa et al. relied solely on traditional CNN architectures, which, although effective in learning local spatial features, do not capture global contextual information essential for dermatological image understanding. Similarly, Sivakumar et al. and Sabir et al. presented pipelines that lack feature fusion from multiple models and overlook the potential of transformer-based networks such as ViTs. Other previous studies, such as Gamage et al. and Abdullah et al., introduced preliminary CNN-ViT integrations. However, these approaches treat CNN and ViT outputs independently and lack a unified feature-level fusion strategy. In contrast, the proposed hybrid model incorporates seamless integration at the feature level, enhancing classification accuracy and generalizability across all lesion classes. Furthermore, while studies by Swetha et al., Kandhro et al., and Adebiyi et al. explored multiple CNNs and multimodal data, they did not optimize deeper feature representations using hybrid techniques or transformer-based attention modules. In contrast, the proposed model EViT-Dens169 addresses these issues through a hybrid architecture that fuses the DenseNet169 CNN with the EViT Encoder model. This fusion enables simultaneous local and global feature extraction—DenseNet169 excels at dense spatial representations, whereas the EViT Encoder captures long-range dependencies via self-attention mechanisms. The superior ability of the proposed model to integrate and balance these complementary features contributes to its robustness and adaptability, particularly in challenging lesion types, such as Dermatofibroma and Vascular Lesions, where prior models underperform. The EViT-Dens169 model had a high AUC (95.17%), accuracy (97.10%), and specificity (99.29%), particularly in underrepresented classes, demonstrating its reliability and clinical relevance.

The novelty and strength of the proposed EViT-Dens169 model lies in its fusion of the CNN and ViT architectures, allowing comprehensive feature extraction and robust classification. It surpasses prior models by addressing class imbalance, enhancing feature diversity, and improving performance across all lesion categories in the ISIC 2018 dataset. The proposed approach represents a critical advancement in the field of intelligent dermatological diagnostics.

The significance of this study lies in the introduction of a novel hybrid deep learning framework that synergistically combines an Enhanced ViT with a computationally optimized DenseNet169 model for the early detection and classification of skin cancer from dermoscopic images. This hybrid model effectively leverages the complementary strengths of CNNs and transformers, namely local spatial feature extraction and global contextual understanding, both of which are critical for precise skin lesion classification. The ViT architecture was improved through the integration of the SDEB and enhancement of MHSA mechanisms. The SDEB acts as a specialized convolutional module that precedes the transformer encoder, recovering the fine-grained spatial continuity lost during patch division. The architecture contains six sequential EViT Encoder blocks, each enhancing depth, positional sensitivity, and representational capacity. In parallel, DenseNet169 was adapted to reduce the computational overhead without compromising feature richness. This was achieved by reducing unnecessary parameter redundancy through selective layer modification and the efficient reuse of features. The fusion representation, obtained by summing the flattened outputs of the DenseNet169 and EViT Encoder branches, was maintained. Both local micro-patterns and global contextual semantics are incorporated to produce an overall feature map that improves the classification accuracy of complex dermoscopic classes.

A significant limitation of this study is the class imbalance in the ISIC 2018 dataset, which may adversely affect model generalization and bias predictions towards the more dominant classes. To overcome this issue, a data augmentation process was applied.

## Conclusions

This study presents a novel hybrid EViT-Dens169 model for dermoscopic image classification that integrates an enhanced ViT mode with an improved DenseNet169 architecture. The EViT Encoder component addresses the spatial discontinuity of traditional ViTs through the SDEB block, which employs multiscale convolutional layers to recover fine-grained local features. The enhanced transformer comprises five EViT Encoder blocks with expanded MHSA mechanisms, enabling robust global context modeling. The DenseNet169 backbone extracts hierarchical local features, whereas the EViT Encoder captures the long-range dependencies. Feature fusion via element-wise addition combines both pathways to optimize the lesion characterization. Evaluation on the ISIC 2018 dataset demonstrated state-of-the-art performance, with an AUC of 95.17%, accuracy of 97.10%, sensitivity of 90.83%, and specificity of 99.29%. The model excels in classifying melanoma (96.6% AUC, 93.8% sensitivity) and nevi (99.7% accuracy), while significantly improving the detection of rare classes, such as dermatofibroma (87% accuracy) and vascular lesions (95.8% precision). The EViT-Dens169 model synergizes local detail preservation and global context modeling, thereby achieving a clinically reliable skin cancer diagnosis.

As part of our future work, we aim to explore the integration of more recent and powerful transformer architectures, such as the Swing Vision Transformer (Swing ViT), with various CNN backbones. This includes adapting and evaluating hybrid models that combine the global feature extraction capabilities of Swing ViT with the local spatial feature learning strengths of the advanced CNNs.

## Data Availability

The dataset used in this study to develop and evaluate the proposed skin cancer classification system is the publicly accessible ISIC 2018 dataset. It can be downloaded from the following repository: https://challenge.isic-archive.com/data/#2018.
